# Blood-based gene expression as non-lethal tool for inferring salinity-habitat history of European eel (*Anguilla anguilla*)

**DOI:** 10.1038/s41598-022-26302-y

**Published:** 2022-12-22

**Authors:** Francesca Bertolini, Mehis Rohtla, Camilla Parzanini, Jonna Tomkiewicz, Caroline M. F. Durif

**Affiliations:** 1 National Institute of Aquatic Resources, Technical University of Denmark, Kongens Lyngby, Denmark; 2 Division of Animal Sciences, Department of Agricultural and Food Sciences, University of Bologna, Bologna, Italy; 3 Institute of Marine Research, Austevoll Research Station, Storebø, Norway; 4 Estonian Marine Institute, University of Tartu, Tartu, Estonia; 5 Department of Nutritional Sciences, University of Toronto, Toronto, Canada

**Keywords:** Functional genomics, Gene expression

## Abstract

The European eel is a facultative catadromous species, meaning that it can skip the freshwater phase or move between marine and freshwater habitats during its continental life stage. Otolith microchemistry, used to determine the habitat use of eel or its salinity history, requires the sacrifice of animals. In this context, blood-based gene expression may represent a non-lethal alternative. In this work, we tested the ability of blood transcriptional profiling to identify the different salinity-habitat histories of European eel. Eels collected from different locations in Norway were classified through otolith microchemistry as freshwater residents (FWR), seawater residents (SWR) or inter-habitat shifters (IHS). We detected 3451 differentially expressed genes from blood by comparing FWR and SWR groups, and then used that subset of genes in a machine learning approach (i.e., random forest) to the extended FWR, SWR, and IHS group. Random forest correctly classified 100% of FWR and SWR and 83% of the IHS using a minimum of 30 genes. The implementation of this non-lethal approach may replace otolith-based microchemistry analysis for the general assessment of life-history tactics in European eels. Overall, this approach is promising for the replacement or reduction of other lethal analyses in determining certain fish traits.

## Introduction

The European eel (*Anguilla anguilla*) is considered a facultative catadromous fish. While it spawns in the ocean, it displays several strategies during its continental growth phase by either growing in freshwater systems, skipping the freshwater phase or shifting between various salinity habitats (i.e., seawater, brackish water, freshwater)^1^. Stock assessment is almost only based on the freshwater contingent of the species^2^. Eels living their entire lifecycle in seawater (i.e., skipping the freshwater phase) are rarely included in assessments and their proportion relative to the whole population is unknown. Moreover, when eels are surveyed, it is unknown whether some have recently shifted between habitats. This distribution pattern complicates stock assessment and management of this species.

Otolith microchemistry is a method commonly applied to determine salinity-history in fish and thereby their use of habitats. Such analysis rely on the variation of ambient water chemistry, as chemical elements are incorporated into the otolith in a predictive manner throughout the life of the fish^1,3^. Otolith microchemical analysis is reliable but requires the sacrifice of the animal. Nonetheless, it is so far the only method which provides migratory information throughout the entire life of a fish.

More recently, fatty acid analysis has also been considered to determine habitat salinity history of eel. The use of fatty acid biomarkers relies on the variation of dietary sources across habitats (in particular between marine and freshwater habitats), which is then reflected in the fatty acid composition of the consumer^4^. This analysis typically requires biopsy of the muscle among other tissues and/or organs (e.g., liver), providing relatively recent (i.e., weeks to several months) feeding information.

In this context, blood-based biomarkers may represent a non-lethal alternative to determine individual salinity-habitat history. Blood is a living tissue that transports molecules throughout the body. A blood sample can, therefore, reflect an individual’s physiological state regarding its health, nutrition, reproductive development, stress and/or metabolism^5–8^. Moreover, blood can be sampled without euthanasia in a high number of species. For identification of possible biomarkers, blood transcriptome is of particular interest, as gene expression is a crucial regulator of cell functions. Here, individual variability, physiological status and external factors, highly influence gene expression^9^. Through the improvement of Next Generation Sequencing (NGS) technologies, and the development of RNA-seq^10^ and bioinformatics tools, it is now possible to simultaneously profile the transcriptional activity of all genes in a desired tissue, from small starting amount of RNA. This has accelerated the discovery of informative genes that can represent biomarkers linked to specific physiological conditions and with a wide range of applications^9,11,12^.

Whole blood transcriptome analysis has been used in several mammalian species, including humans, to identify biomarker indicators of pathological conditions, immune competences, and reproductive performance^13–17^. In mammalian blood, the only nucleated and hence transcriptionally active cells are the white cells (i.e., leucocytes). In contrast, red blood cells (i.e., erythrocytes) of non-mammalian vertebrates are also nucleated and hence transcriptionally active^18^. This makes non-mammalian blood potentially more informative than mammalian blood.

The recent and limited number of studies targeting blood transcriptomics in non-mammalian vertebrates, such as birds and reptiles, show that genetic activity between blood and liver have a high degree of similarity, especially in terms of biological processes^19,20^. The liver is an organ with high functional gene expression diversity, and it is frequently used in transcriptomic investigations. Blood transcriptomic-based analyses show applications in ecology, ecophysiology, and toxicology studies where gene transcripts in the blood have been studied as candidate indicators of behavior, physiological condition, environmental impacts and other phenotypic differences, particularly for wild species ^20–23^.

Similar outcomes have been produced in the even more limited number of studies in fish, both in experimental and in field settings. A study on fathead minnow *Pimephales promelas,* investigating exposure to environmentally relevant concentrations of chemical substances, showed how blood responded with a greater number of altered genes compared to liver, and how they shared the same biological altered pathways^24^. Another study detected 563 blood transcript biomarkers that can be used for non-lethal sex differentiation in the Asian swamp eel *Monopterus albus*^25^.

The aim of the present study was to investigate whether whole blood transcriptional profile could be used to infer the salinity-habitat history from eels collected in the wild. To do this, we sampled blood, otoliths, and muscle tissue from European eels collected in different salinity habitats to compare transcriptomes with the otolith microchemistry (entire life) and fatty acid profiles (recent migratory history), which served to validate individual eel salinity histories. We identified candidate genes that could be used as transcriptomic-based biomarkers to discriminate salinity-habitat history and applied a machine learning approach to evaluate the ability of those biomarkers to match the classification derived by otolith microchemistry analysis. In recent years, machine learning algorithms have been used for the analysis of high-throughput deep sequencing data due to their computational efficiency in finding generalizable patterns from high-dimensional data obtained from a small number of samples^26^. Here, we used random forest (RF), a supervised machine learning algorithm that is widely used in classification and regression problems^27^.

## Material and methods

### Statement

Sampling and handling of eels in this study were approved by the Norwegian Animal Research Authority and all procedures followed local animal welfare regulations (FOTS id 15952) and are in accordance with ARRIVE guidelines.

### Samples collection

**Figure 1:**
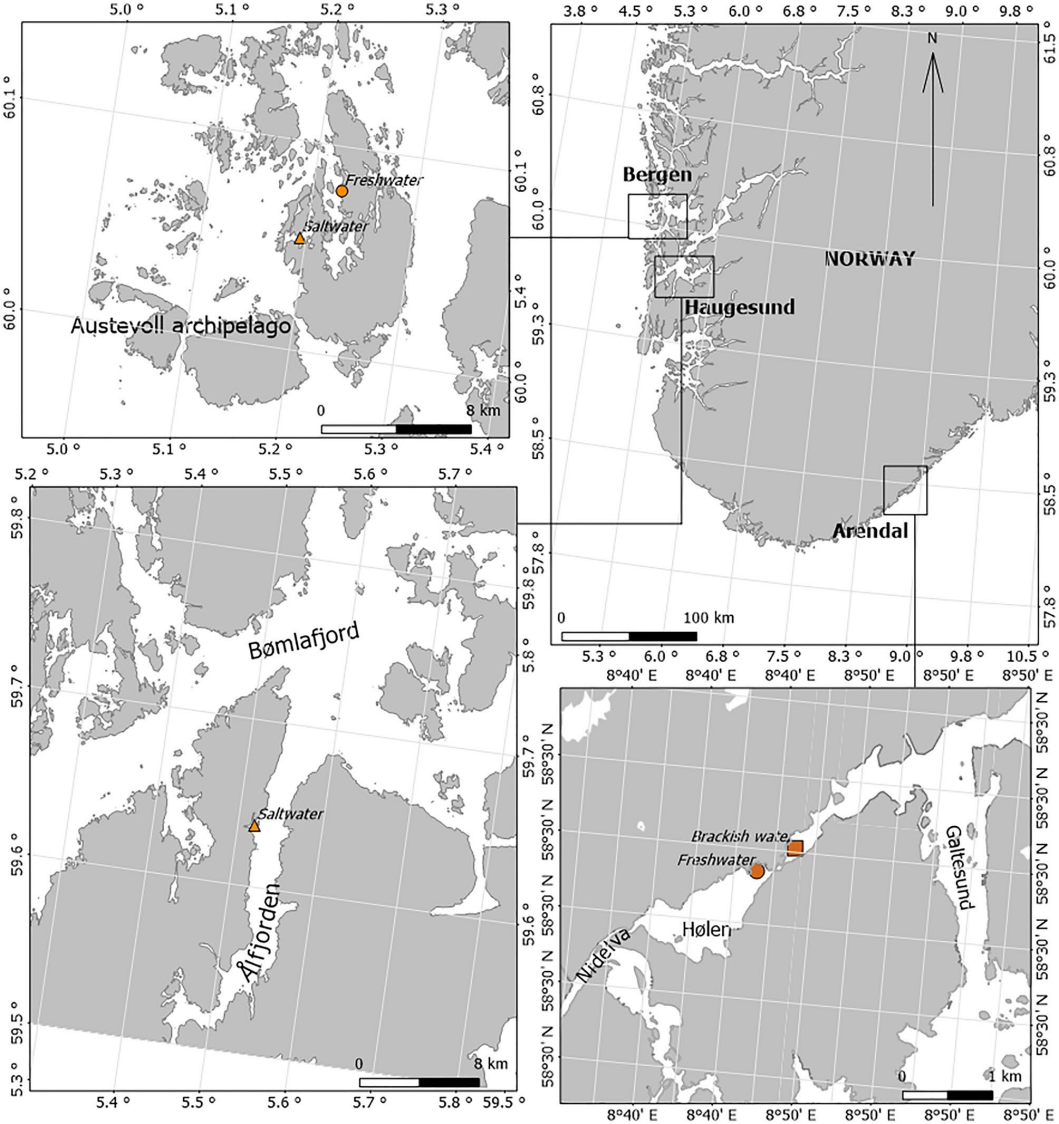
Map of the Norwegian locations of European eel sampling.

Eels were collected in July and August 2020, in five sites in Norway which represented three different salinity habitats (Fig. 1): freshwater (FW; Arendal and Bergen), brackish water (BW; Arendal), and seawater (SW; Bergen and Haugesund). Eels were caught using fyke nets (mesh size at the cod end was ∼8 mm, knot-to-knot, and 11 mm along the diagonal) and eel pots (mesh size was ∼ 10 mm, knot-to-knot, and 15 mm diagonal). Brackish waters are often characterized by salinity stratification making it difficult to assign an exact salinity value to each location. Therefore, we considered SW sites to correspond to salinities > 30 ppt, BW to comprise sites corresponding to salinities between 0.5–30 ppt, and FW sites to be < 0.5 ppt.

A total of 151 individuals were captured and anaesthetized with clove oil and measured for total length (mm), wet mass (g), eye diameter (mm) and fin length (mm). These measurements were used to assign a silvering stage, where stages I and II represent eels in their growth phase (classic “yellow” phase), stage III indicates a pre-migrant phase, and stages IV and V are the two migratory “silver” phases^28^.

From 60 anaesthetized animals, approximately 600 µl of blood was collected in lithium heparine tubes, mixed with 2 volumes of RNA-later (Invitrogen) and stored at − 20 °C prior to RNA extraction. Anaesthetized eels were then euthanized and dissected. The sex of all captured eels was determined by macroscopic observation of gonads. Skinless, white muscle tissue close to the dorsal fin (∼ 1.0 × 0.5 cm) was sampled for fatty acid analysis. Sagittal otoliths were dissected for microchemistry analysis (Table S1).

### Otolith and fatty acid-based classification

Transversal otolith thin sections were prepared for chemical analysis of otoliths. Otolith thin sections were analysed for ^24^ Mg, ^43^Ca, ^55^Mn, ^88^Sr and ^137^Ba using laser ablation inductively coupled plasma mass spectrometry at the University of Tartu (Department of Geology). A continuous line scan was traced from the core to the edge using a laser speed of 5 µm/s and laser beam diameter of 40 µm. Eels were classified as seawater residents (SWR), freshwater residents (FWR) or inter-habitat shifters (IHS) based on established otolith freshwater reference values of Sr:Ca and Ba:Ca (see Rohtla et al.^29^ for additional details on chemical analysis and data interpretation).

Lipids were extracted using a modified version of the Folch et al.^30^ method in a chloroform:methanol (2:1) solution, and fatty acids analyzed as methyl esters (FAME) through gas-chromatography and flame ionization detection (GC-FID) at the Department of Chemistry and Biology of Ryerson University, Toronto, Canada. Individual fatty acids were identified using a series of standards, including a 39-component FAME mix (GLC-463, Nu-Check Prep Inc.), a marine PUFA mix inclusive of 22:1n-11 (11-docosenoic acid methyl ester; PUFA Mix No.1, Supelco Inc.), and 18:4n-3 (stearidonic acid; SMB00291, Sigma-Aldrich). The M/F ratio was used to determine eel diet^31^ This ratio provides information on a largely marine-(M; higher values) versus freshwater-based diet (F; lower values), and hence, indirectly, information on the feeding habitat (i.e., SW, FW), depending on the presence of a few characteristic fatty acids in the muscle tissue. Intermediate values were assumed to represent eels feeding and living in BW and/or eels regularly moving between SW and FW.

### RNA extraction and sequencing and data mining

RNA extraction was performed following a modified Trizol (Invitrogen) protocol, starting from 200 µl of mixed whole blood + RNA-later. RNA integrity was assessed with Bioanalyzer, to estimate the RNA Integrity Number (RIN) for each of the samples. All samples had RIN > 8 and were therefore evaluated as intact and suitable for sequencing. Paired end mRNA-seq 150 bp was performed at Novogene co (China).

Read quality was assessed by Fastqc^32^. Reads were trimmed with Trimmomatic v0.38^33^, removing the first 9 bases at the beginning of the reads, as well as part of reads that had lower quality and reads that were shorter than 36 bp after trimming (HEADCROP:9, SLIDINGWINDOW: 4:15, MINLEN:36). Mapping of trimmed reads was performed with Tophat2 v0.13^34^ with default options using the latest version of the European eel reference genome and annotation fAngAng1.pri (NCBI; GCF_013347855.1) to guide the read mapping. A further filter was performed with Samtools v1.10^35^, removing reads that mapped in multiple places and sorting the reads by read name, as condition to run the reads count. For every samples, reads count at each annotated gene was performed with htseq-count^36^.

### Differential expression and gene enrichment

Transcriptome analysis was performed at first with the overall dataset using the Deseq2 R package^37^ considering the normalized log2-fold change. Differential expression was then done with the same package considering only FWR and SWR derived from the otolith analyses. Here, genes with adj *p* values < 0.05 were considered as significantly differentially expressed (DE) and used for subsequent gene enrichment and Random Forest (RF) analyses.

Gene enrichment was performed with upregulated and downregulated genes separately using Panther (http://www.pantherdb.org/), considering Gene Ontology (GO) biological processes as annotation set and *Danio rerio* as reference gene set. Only GO terms with FDR *p* < 0.05 were considered. Redundant GO terms were removed with REVIGO^38^, utilizing the *Danio rerio* database and applying SimRel as semantic similarity measure.

### Random forest

In a classification context, RF allows to assign a unit for which the class is unknown to a pre-determined group, using the so-called majority rule to aggregate the ‘B’ predictions obtained from the different trees in the forest. The final predicted class is the most commonly occurring one. In RF, as trees are fitted to bootstrapped subsets of the data set, some observations are left aside each time a tree is built. This leads to a valid estimate of the prediction error of a random forest, which is the so-called out-of-bag (OOB) error^27^. Predictors can then be ranked according to their relevance in the classification rule in two measures: the Mean Decrease in the Gini Index and the Mean Accuracy Decrease^27^.

RF on the reduced gene set was performed over normalized gene expression data, obtained through a log-transformation of the whole gene set with Deseq2^37^. Then, the log-transformed values of the DE genes were used for the RF analysis considering the whole sample set. Samples were classified according to the otolith analyses into three groups: FWR, SWR, and IHS. RF analysis was performed with the R package randomForest^39^, with 700 trees. Each analysis was repeated 10 times and the average values were calculated. From the first round of analyses with all the DE genes, Mean Decrease in the Gini Index and the Mean Accuracy Decrease were also extracted. Then, genes with no contribution (= 0) to Gini and Accuracy in at least one of the groups (i.e., FWR, IHS, SWR) were removed. The remaining genes were sorted according to their Mean Decrease in the Gini Index or Mean Accuracy Decrease. RF was then run again with the same parameters for ten times each, to assess the OOB and the classification error of panels using the top 150, 100, 50 and 30 genes based on their Mean Decrease in the Gini Index and the Mean Accuracy Decrease values.

## Results

### Comparison between transcriptional profiling, otolith microchemistry and fatty acid profiles

Among the 60 eels included in this study, 26 individuals were captured in SW, 7 in BW, and 27 in FW. Otolith analysis classified 11 of these eels as FWR, 23 as IHS, and 25 eels as SWR, and 1 sample was unclear (SWR/IHS). The fatty acid-based classification reported 19 animals as from FW, 19 as BW and 13 as SW, while the habitat of 8 eels remained undecided, and 1 not classified (Table S1).

The RNA-seq data production and subsequent trimming yielded 43,008,662 ± 3,559,138 high-quality reads used for mapping. Approximately 76.71% ± 2.69 of the reads were successfully mapped to the reference genome (Table S1).

**Figure 2:**
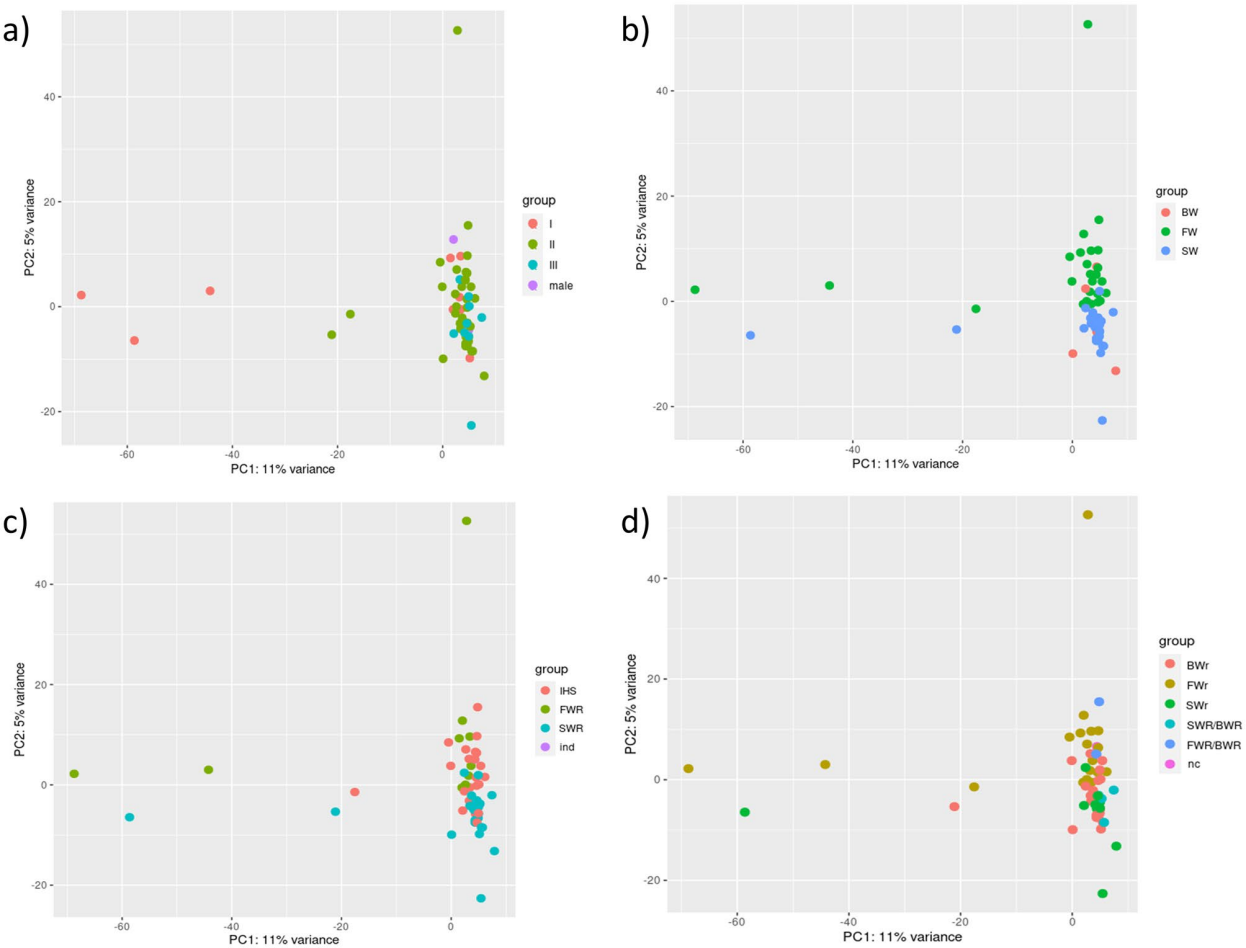
Principal component analysis (PCA) of the samples based on log2transformed transcriptomic values labelled based on their sex, that is silvering stage for female (I, II, III) and male (**a**), sampling site (**b**), otolith-based history (**c**) and fatty-acid based history (**d**).

Figure 2 shows the principal component analysis (PCA) based on the blood transcriptomic analysis, where 2 outliers were omitted. Sample clusterization by silvering stage did not show any evident grouping (Fig. 2a), and this factor was hence not considered a main driver of variation in this analysis. In contrast, sample clusterization by salinity habitat provided a clearer picture, with SW and FW eels occupying two close but distinctive clusters on the y-axis, and BW eels encompassing these clusters (Fig. 2b). Labelling according to the results from the otolith analysis also highlighted differences between FWR and SWR eels, with IHS samples plotted between the two habitats (Fig. 2c). Labelling according to fatty acid profile was concordant with the results provided by the previous classifications (Fig. 2d). The uncertain individuals were closer to the “FW” area of the plot for the FW/BW and closer to the SW area for the SW/BW. Altogether, habitat salinity was a major driver for the clusterization of the transcriptomic profile, and otolith-based classification produced the lowest number of uncertainties in the classification, as well as a clear division between SWR and FWR.

### Differential gene expression between salinity habitats

**Figure 3:**
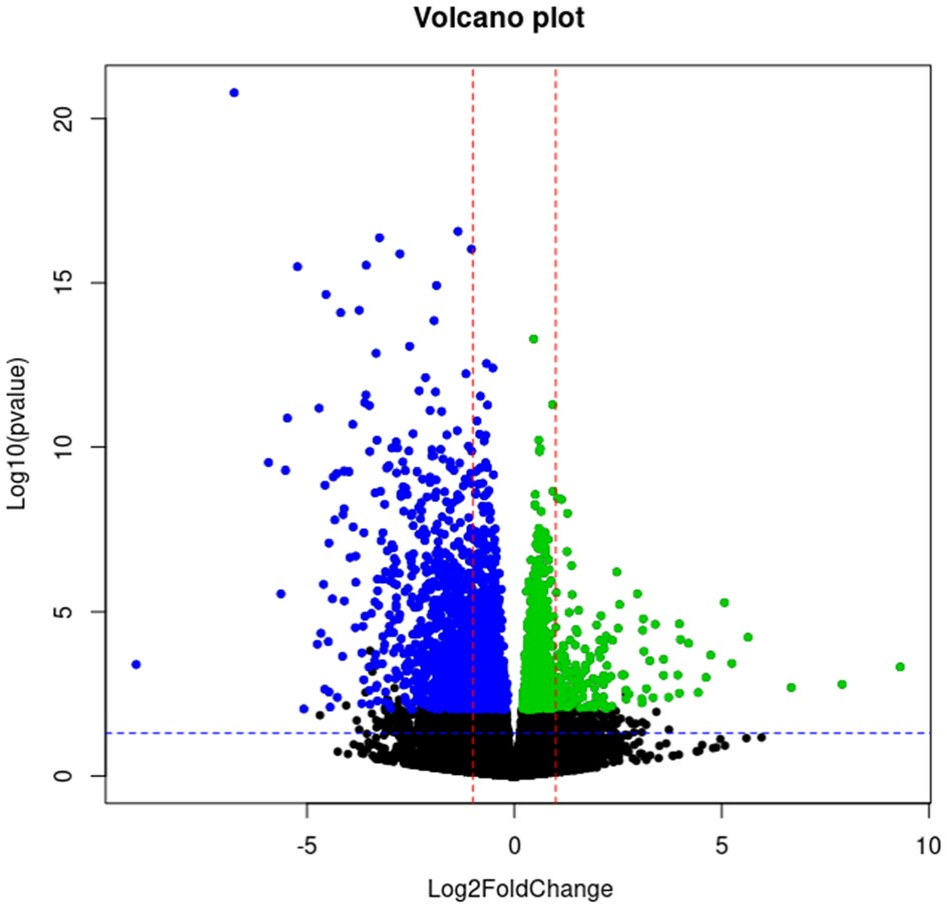
Distribution of differentially expressed genes, with genes more expressed in freshwater resident eels (blue) and genes more expressed in seawater resident eels (green). Black dots represents non-significant differentially expressed genes. X-axis reports the intensity of gene expression change expressed in Log2fold change.

DE was calculated comparing FWR and SWR eels. The analysis detected 3451 genes with adj*p*value < 0.05. Among these, 1496 genes were upregulated when eels were classified as SWR and 1955 genes were upregulated when eels were classified as FWR. Four genes were removed from the analysis, as they were outliers based on visual inspection of the plot distribution (Fig. 3, table s2).

**Figure 4:**
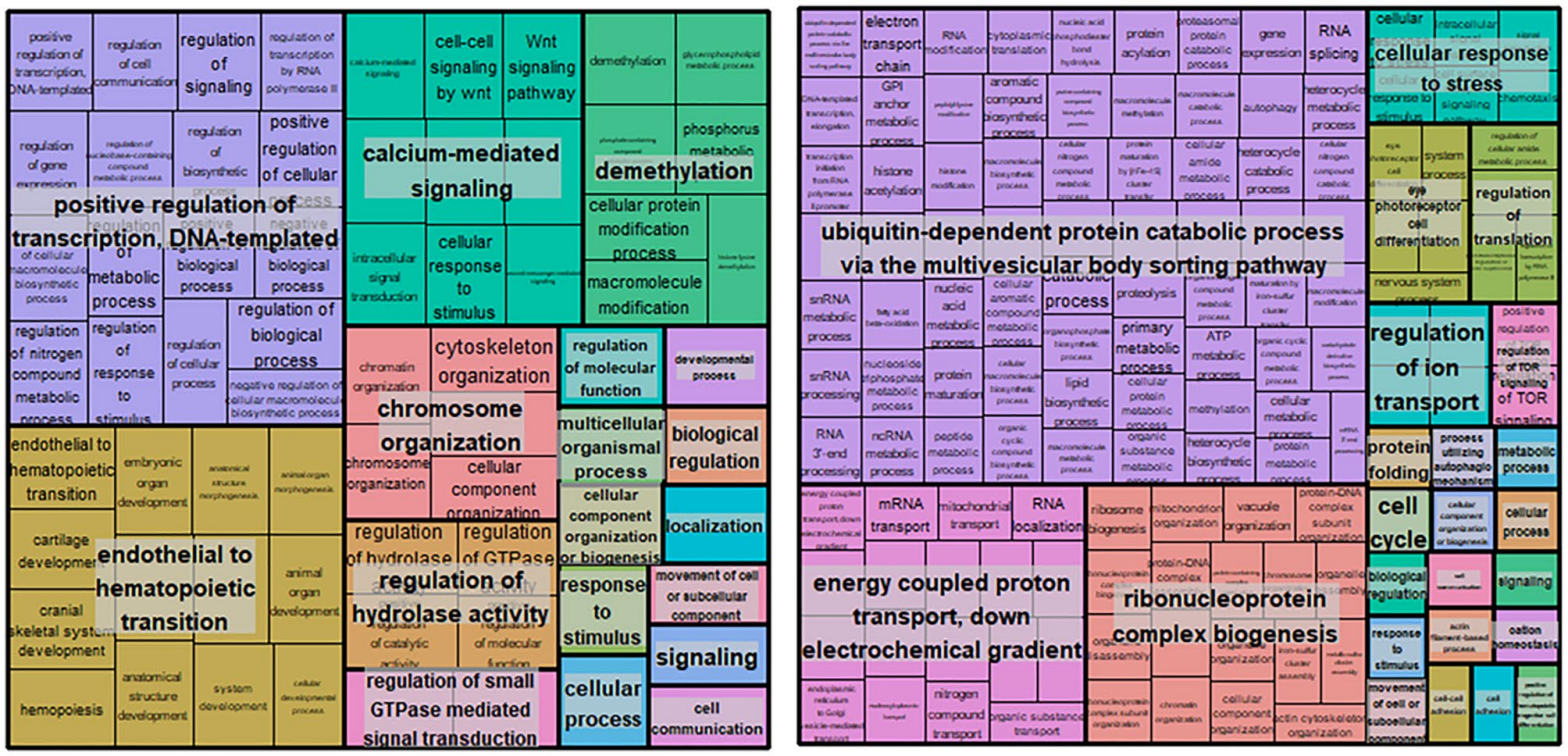
Treemap of the downregulated GO term (left) and upregulated Goterm (right). The nested rectangles, representing the branches of the tree, are not scaled. See Tables S3 and S4 for *p*-values and fold changes, as well as details of the names and genes.

Enrichment analyses reported 143 enriched Gene Ontology (GO) biological terms for the genes more expressed among FWR, and 250 for genes that were more expressed among SWR. After removing redundant terms 76 GO terms for downregulated and 143 Go terms for the upregulated were retained (Tables S3 and S4). A total of 19 GO terms overlapped between the two gene sets, and this may indicate common pathways between the two environments (i.e., different genes involved in the same pathways). These terms are mainly related to regulation and organization of basic cells components, particularly RNA expression (e.g., signal transduction, regulation of transcription by RNA polymerase II, organelle organization, chromosome organization). Most GO terms revealed distinct pathways that may be activated in different salinity environments. In FWR, GO terms related with developmental processes, morphogenesis and immune functions (e.g., cranial skeletal system development, cartilage development, hematopoiesis, Wnt signaling and immune system development; Fig. 4 and Table S3). In SWR, the most relevant processes were linked to ATP production and ion transport, response to stress and fatty-acid oxidation (e.g., energy-coupled proton transport, electrochemical gradient, ATP metabolic process, electron transport chain, mitochondrial transport, fatty acid beta-oxidation; Fig. 4 and Table S4).

**Figure 5:**
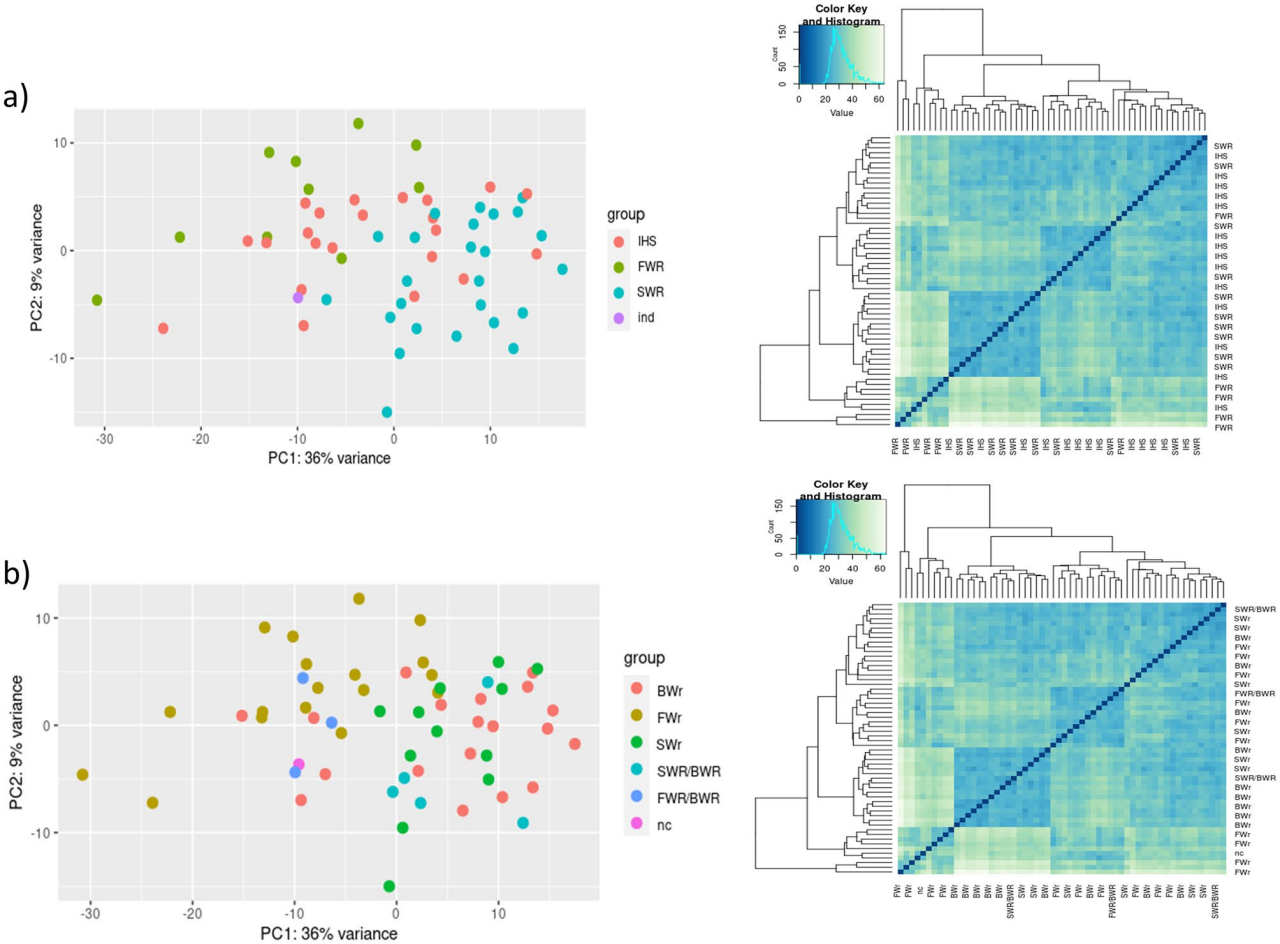
Principal component analysis (PCA) (left) and heatmap (right) for the otolith-based (**a**) and fatty acid (**b**) analyses based on the DE genes. For the heatmap, the intensity of the color is linked with the similarity of the sample in pairwise comparisons, where light color indicates higher divergence and darker color indicates higher similarity.

Repeated PCA based plot considering only the DE genes, but expanded on the whole sample set, enhanced the separation between salinity profiles (Fig. 5). The division was particularly clear for the otolith-based analysis, where the DE genes were detected (Fig. 5a). There, the only sample that could not be classified in the otolith analysis (labelled as “ind”) was classified as IHS after the blood transcriptome-based positioning in the PCA plot. Despite being less prominent, a gradient was also detected in samples classified according to their lipid profiles. There, it was more evident that the eels with intermediate values of the M/F ratio (i.e., FW/BW and SW/BW) tended to be closer to the FW and SW part of the plot respectively (Fig. 5b).

### Classification of eel into salinity habitats using random forest

RF classification, using the complete set of 3451 DE genes, correctly classified 79% of the eels into their salinity habitat (average OOB = 21%). Mis-assignments concerned mostly FWR and IHS eel (Table 1). The subset of genes further selected considering both Mean Decrease in the Gini Index and Mean Accuracy ranking improved the overall results, especially in the correct assignment of FWR individuals. For both ranking categories, correct classification increased by retaining only the top ranked genes until reaching a maximum value of rate of 93.1% (OOB = 6.9%) for both rankings (Table 1). The results were quite similar, with 100% of correctly assigned SWR and FWR (CCP FWR = 1 and CCP SWR = 1) in all the four subsets (150-100-50 and 30 genes) and only four misplaced animals belonging to IHS in the 50 and 30 genes panels (CCP IHS = 0.84; Table 1). Misclassified samples were B121, B128, B173 and B181 which were classified as IHS by otolith analyses, but as SWR by RF, reflecting the recent salinity habitat, (i.e., capture location in SW). This overlap in the results derives also from the fact that most genes, at least in the 30 genes panels (93% for the 30 gene panels), overlapped between the two rankings (Table 2). Among the top 30 genes for mean decrease Gini and mean accuracy, 10 genes (*mctp2a, inppl1b, asap1b, itk, adra1ba, rerea, tead1b, nelfb, acin1a* and *rev1*) are included in one or more biological processes previously detected in the enrichment analysis. The processes were mainly related to general metabolic and regulation processes, or developmental processes, hematopoiesis and immune system (Table S5).

**Table 1:** OOB error rate (%) and the CPP of the different gene sets: all differentially expressed, and top 150, 100, 50 and 30 bases on Mean Decrease in the Gini and Mean Accuracy Decrease. CCP = (1-classification error).

	All DE	Mean decrease in the gini				Mean accuracy decrease			
		150	100	50	30	150	100	50	30
OOB error rate (%)	18.97	8.62	8.62	6.9	6.9	8.62	8.62	6.9	6.9
CCP FWR	0.80	1.00	1.00	1.00	1.00	1.00	1.00	1.00	1.00
CCP SWR	0.96	1.00	1.00	1.00	1.00	1.00	1.00	1.00	1.00
CCP IHS	0.67	0.80	0.80	0.84	0.84	0.80	0.80	0.84	0.84

**Table 2:** Genes included in the two 30-gene panel for random forest classification.

Gene symbol	Gene name	chromosome	MeanDecreaseAccuracy	MeanDecreaseGini
rasal1	rasGAP-activating-like protein 1	NC_049210.1	0.0083	0.4798
mctp2a	Multiple C2 domains, transmembrane 2a	NC_049216.1	0.0041	0.2491
LOC118211351	Zinc finger E-box-binding homeobox 2-like	NC_049213.1	0.0033	0.2325
rnf138	E3 ubiquitin-protein ligase RNF138	NC_049204.1	0.0034	0.2300
LOC118227625	paired box protein Pax-5-like	NC_049205.1	0.0037	0.2274
fgd6	FYVE, RhoGEF and PH domain containing 6	NC_049207.1	0.0028	0.2197
inppl1b	Nositol polyphosphate phosphatase-like 1b	NC_049203.1	0.0034	0.2165
hic2	Hypermethylated in cancer 2 protein-like	NC_049210.1	0.0024	0.2131
LOC118231111	Osteoclast stimulatory transmembrane protein-like	NC_049207.1	0.0027	0.2094
LOC118208373	filamin-A-like	NC_049202.1	0.0034	0.2013
LOC118219836	CCN family member 1-like	NC_049211.1	0.0026	0.1967
LOC118225135	cadherin-24-like	NC_049204.1	0.0026	0.1909
LOC118229298	C–C motif chemokine 20-like	NC_049206.1	0.0025	0.1892
LOC118235156	uncharacterized LOC118235156	NC_049209.1	0.0023	0.1702
asap1b	ArfGAP with SH3 domain, ankyrin repeat and PH domain 1b	NC_049204.1	0.0021	0.1660
LOC118226320	Nuclear factor interleukin-3-regulated protein-like	NC_049204.1	0.0014	0.1520
Itk	IL2 inducible T cell kinase	NC_049203.1	0.0018	0.1493
adra1ba	adrenoceptor alpha 1Ba	NC_049209.1	0.0018	0.1461
LOC118233432	Dynein regulatory complex protein 11-like	NC_049208.1	0.0018	0.1420
Rerea	Arginine-glutamic acid dipeptide (RE) repeats a	NC_049213.1	0.0016	0.1390
tead1b	TEA domain family member 1b	NC_049203.1	0.0015	0.1293
hivep3	Transcription factor HIVEP3	NC_049201.1	0.0013	0.1276
LOC118235830	myelin-associated glycoprotein-like	NC_049201.1	0.0017	0.1271
LOC118211187	forkhead box protein J3-like	NC_049213.1	0.0016	0.1265
nelfb	Negative elongation factor complex member B	NC_049210.1	0.0012	0.1190
acin1a	Apoptotic chromatin condensation inducer 1a	NC_049208.1	0.0013	0.1189
mob3b*	mob kinase activator 3b	NC_049214.1	0.0011	0.1158
zap70	tyrosine-protein kinase ZAP-70	NC_049204.1	0.0013	0.1150
rev1*	REV1 DNA directed polymerase	NC_049215.1	0.0012	0.1143
LOC118229170	isthmin-2-like	NC_049206.1	0.0013	0.1113
agpat4^+^	1-acylglycerol-3-phosphate O-acyltransferase 4 (lysophosphatidic acid acyltransferase, delta)	NC_049218.1	0.0013	0.1046
epb41l1^+^	Band 4.1-like protein 1	NC_049213.1	0.0012	0.0995

*Gene unique for the 30 top gene panel based on gini values.

^+^Gene unique for the 30 top gene panel based on accuracy values.

## Discussion

The salinity-habitat history of European eel can be determined with reasonable accuracy using whole blood transcriptomic analysis. This method can replace or reduce lethal-based assessments (e.g., using otolith microchemistry, fatty acid analysis). This is relevant for fish species in general, but more specifically for sensitive and/or (critically) endangered species, such as the European eel.

In the present study, RNA-seq performed on small blood amounts of 60 adult eels (58 after removing of outliers), collected in different salinity habitats (i.e., FW, SW, and BW) was first assessed through PCA plots based on the normalized transcriptomic count in relation to sampling location, otolith-based classification, fatty acid-based analysis, and to other external drivers linked to reproductive traits that can influence transcriptomics (i.e., silvering stage and sex). Here, we observed a pattern driven by salinity, independently of the means of classification (i.e., collection site, otolith microchemistry, or fatty acid profiles) in contrast to silvering stage. This clusterization was observed when considering a reduced 3451 DE gene dataset, which was then used for machine learning analysis. The number of studies investigating blood transcriptomics in non-mammals is very limited, and hence there is no map of candidate genes that could be affected by salinity changes in whole blood.

### Physiology related to salinity habitats

Investigations on the adaptation of teleost fish to different salinity conditions have focused on gill and gut epithelium. In these tissues, hypersalinity (i.e., > 35 ppt) leads to an increase of Na^+^, K^+^-dependent ATPase activities^40–42^. In plasma, Na^+^ and Cl^−^ ion levels increase in euryhaline species^41^. In our study, SWR eels showed a higher expression of genes related to ATP production (“mitochondrial ATP synthesis coupled electron transport”, “ATP synthesis coupled electron transport”, “ATP synthesis coupled proton transport”, “ATP biosynthetic process”). Therefore, at least in the blood, the activity seems to go in the opposite direction—expressing genes that are involved in ATP production, particularly ATP synthase genes (atp5f1e, atp5l, atp5mc3a, atp5mea, atp5mf, atp5pd, atp5pf) and NADH dehydrogenase (ndufa10, ndufa7, ndufa8, ndufaf1, ndufb8, ndufc2, ndufs2, ndufs6, ndufv3). Ion transport and homeostasis is in line with the plasma of other tissues, with genes related to ion homeostasis, and regulation pathways that are significantly enriched in SWR.

In European eel, diet and forage habitat may represent a major driver of variation in the fatty acid composition of eel muscle tissue^4^. In fact, fatty acid-based analysis has a high discriminating power across salinity habitats. This is also valid regarding blood when incorporating transcriptomic analysis, even with a lower power than otolith-based clusterization. Although not yet investigated in fish, erythrocyte fatty acid composition is considered one of the most stable biomarker for assessing long-term dietary intake or endogenous biosynthesis and metabolism, from several weeks to months prior, in humans^43^. In this study, genes related to fatty acid oxidation and fatty acid beta-oxidation biological processes were more largely represented in the SWR group. Fatty acid oxidation is an important process for ATP production and/or fatty acid storage in muscle/adipose tissue. Indeed, at the same age SW eels were larger than FW eels^29^. Fatty acid oxidation that mainly occurs in the mitochondria, involves a repeated sequence of reactions that result in the conversion of fatty acids to acetyl-CoA^44,45^. This is concordant with what was observed in our data, where ATP synthesis is also upregulated in the SWR. In mammals, erythrocytes do not oxidize fatty acids because they lack mitochondrial or aerobic metabolism, thus relying on cytosolic energy generation instead. As for fish, even if there are reductions in biosynthetic processes and in the ability to mount heat shock responses with cell aging (common in other vertebrates), erythrocytes of fish do not appear to lose functions like the capacity to perform ion and gas transport, as total protein concentration in young red blood cells is not affected by cell age^46,47^.

Osmoregulation is one of the most energetically costly metabolic activities in teleost, as both sea- and freshwater deviate from the salinity of fish body fluids^48^. Therefore, a large amount of energy is consumed by fish to maintain their osmotic homeostasis during acclimation to either freshwater or hyper-saline water^49,50^. At a molecular level, this can have an impact on fish development, growth, reproduction, and other physiological and metabolic activities^50^. This is in line with the high number of identified GO terms that are linked to development and morphogenesis and to phenotypical observation of European eels, where size was bigger in SWR eels compared to FWR and IHS at the same age^4,29^. About 300 genes that are linked to morphogenesis and development were upregulated in FWR and not in SWR. Since development is a complex matter that requires activation and suppression of several pathways, at the present state, it is not possible to provide a more detailed description of these pathways. To be noted is the presence of biological processes related to the immune system development and regulation among the pathways, which were enhanced in FWR. Variations in water salinity may impact the immune function of fish^51,52^. Here, the GO term “cell–cell signaling by wnt” was detected. The Wnt signaling pathway is critical for adult tissue maintenance, remodeling and regeneration, embryo development, and many cellular processes that include cell motility and cytoskeleton restructuring^53^. The Wnt signaling pathway is associated with cellular remodeling in fishes, and it may play a role in salinity adaptation^54,55^. The Wnt signaling also regulates the progenitor cell homeostasis, hereby controlling hematopoiesis that was also upregulated in the FWR group. Even if two of the major genes involved in this pathway (i.e. cdc42 and wnt11) were not significantly differentially expressed, other genes such as ccdc88c, tet2, tet3 and lgr4, which have a known function in this pathway, were differentially expressed in our study^56–58^.

### Biomarkers for classification of salinity-life history

**Figure 6:**
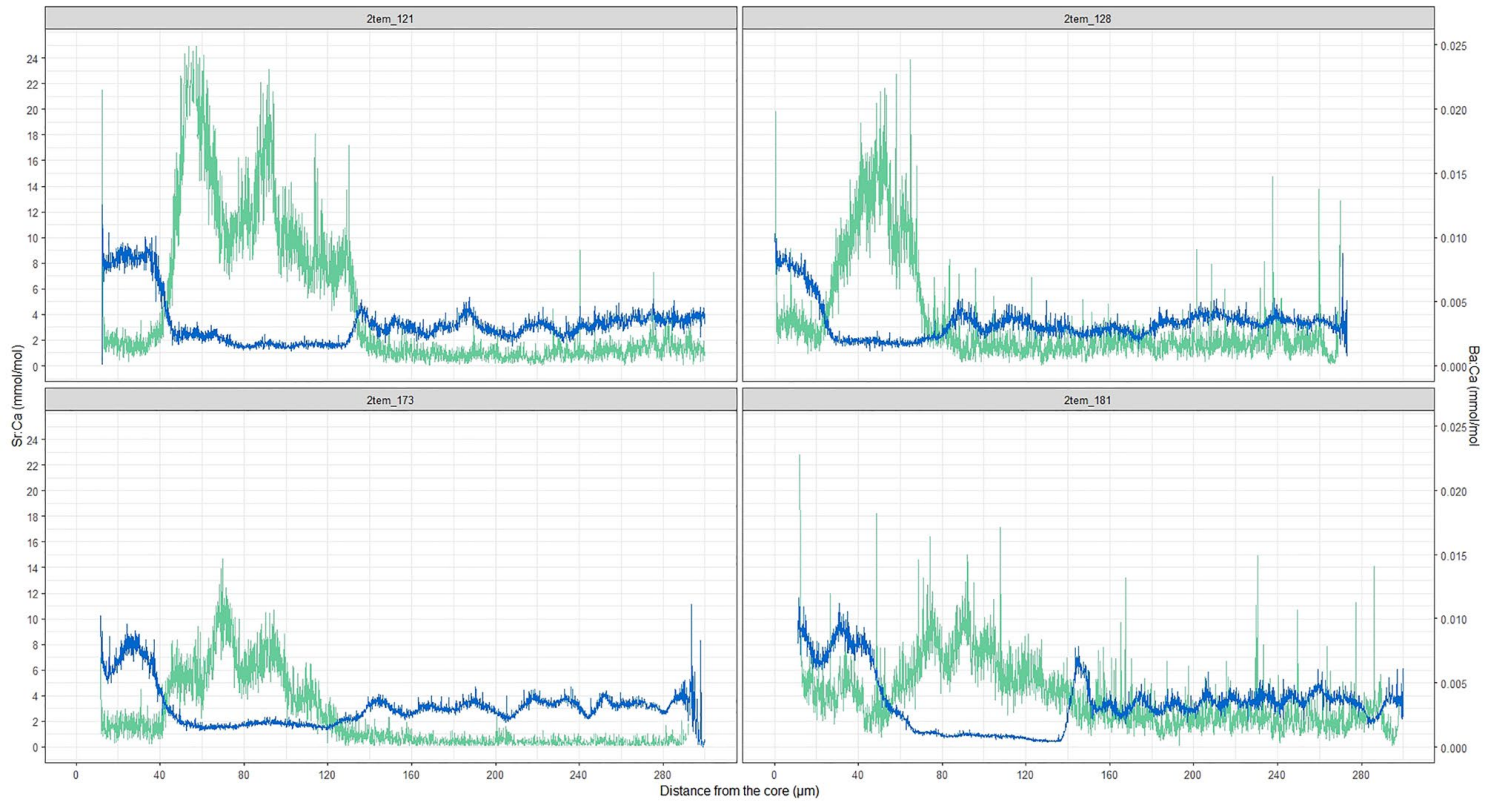
Otolith Sr:Ca (blue) and Ba:Ca (green) profiles of the four inter-habitat shifting eels (*Anguilla anguilla*) misclassified by the random forest analysis on blood transcriptomes.

RF performed using the DE genes as first list, and then reduced using different rankings allowed the correct assignment of all eel samples (except four) into their salinity habitat. The misclassification of these four samples was detected in all gene panels and were classified as IHS by otolith analysis and as BW by fatty acid analysis. Particularly, otolith analysis detected that those misclassified samples had early FW experience (Fig. 6). Otolith analysis can further characterize IHS, by estimating when and how many times, shifts between FW and SW habitats have occurred^29^. This indicates that transcriptomics analysis may fail to show very early FW experience. However, in order to clarify this, more subcategories among the IHS group to be run with RF would be needed. Despite RF tolerating low sample numbers, subcategories of IHS group were not performed in RF analysis due to our relatively limited sample size.

Machine learning is useful to infer phenotypes to samples whose phenotype is unknown or not given in the analysis (i.e., test population) based on information collected from samples with the available phenotype(s) of interest (i.e., training population). In order to maximize the usage of our samples in this work, we did not split our dataset into a training and test. In fact, RF uses bootstrap to create random permutation in the data that leads to the definition of an OOB population for each individual tree in the forest. The OOB population consists of all the samples that are not included in the bootstrap population used to build a given single tree and that can be used to obtain internal unbiased estimates of the prediction error and to evaluate variable importance^39^. The ability of transcriptomic-based biomarkers to distinguish with a relatively high level of accuracy (75%) eels with habitat-shifting history (IHS) from residents (FWR or SWR) regardless of the salinity at the sampling site suggests that salinity habitat history may leave a fingerprint in blood transcriptomics. This could hypothetically be through e.g., epigenetic mechanisms; however, further studies would be needed to decipher this aspect. In this context, controlled laboratory studies might help to identify how long these transcriptional signals reflecting different environments last.

## Conclusions

Our investigation showed that blood transcriptional profile is influenced by salinity habitat history. The combination of machine learning and transcriptomic profiling allowed the assessment of salinity-habitat history with high accuracy, including habitat shifting behaviors. As habitat shifting behaviors may involve several shifts throughout the eel’s continental life, additional studies will be needed to assess the extent of the prediction power of blood transcriptomics. Still, for complete reconstructions of IHS chronological salinity history, otolith microchemistry seems inevitable. However, with an adequate increase and maintenance of the training population, using otolith microchemistry analysis as known phenotype, there may be the possibility to greatly reduce, and in the future substitute, otolith microchemistry analysis for general estimation of fish salinity history. This approach is promising for the replacement or reduction of other lethal analyses in fish research, especially for critically endangered species, such as the European eel. Collecting blood and determining salinity-habitat history during annual monitoring surveys would provide important information improving management of this species. Overall knowledge on eel habitat use, such as migratory season and proportion of individuals that shift habitat, should be considered in the species monitoring. This could highlight the importance of this contingent (in terms of numbers) and the need to improve migratory conditions and habitat availability.

## Supplementary Information

Supplementary Legends.

Supplementary Table S1.

Supplementary Table S2.

Supplementary Table S3.

Supplementary Table S4.

Supplementary Table S5.

## Data Availability

Raw RNA-seq data are available on EMBL-EBI under Accession Number PRJEB52485.
