# Supplementary material for: Blood-based gene expression as non-lethal tool for inferring salinity-habitat history of European eel (Anguilla anguilla)
Source: Sci Rep. 2022 Dec 22;12:22142. doi: 10.1038/s41598-022-26302-y (PMC9780358; doi:10.1038/s41598-022-26302-y)
Supplement: Supplementary file 1 — Supplementary Legends. [file 41598_2022_26302_MOESM1_ESM.docx]

**Blood-based gene expression as non-lethal tool for inferring salinity-habitat history of European eel (*Anguilla anguilla*)**

Francesca Bertolini, Mehis Rohtla, Camilla Parzanini, Jonna Tomkiewicz, Caroline M.F. Durif

**Supplementary information**

Table S1: Samples investigated, classification based on the location at collection (“salinity collection”), otolith analysis (“omc life history”) and fatty acid analysis (“Fatty acids”). Silvering stage for females (I to V) is reported, Total number of sequenced reads and % of reads mapped to the European eel reference genome are also reported. Samples removed from the analyses because considered as outliers are marked with the “*” symbol in the “EEL_ID” column. (see excel).

Table S2: Complete list of significant differentially expressed genes (adjp<0.05), where gene symbol, intensity of the differentially expression and adjusted pvalue is reported (see excel).

Table S3: GO terms biological processes related to the genes more expressed in animals classified as freshwater residents FWR (complete list), including the genes symbol from the Differential expression analysis (see excel).

Table S4: GO terms biological processes related to the genes more expressed in animals classified as seawater residents SWR (complete list), including the genes symbol from the Differential expression analysis (see excel).

Table S5: Enriched GO terms in which the top30 detected transcript for random forest are included (See excel).
